# A Cross-Sectional Study for the Spectrum of Clinical Diagnosis in Patients Presenting With Macrocytosis

**DOI:** 10.7759/cureus.54702

**Published:** 2024-02-22

**Authors:** Syeda Samia Shafaat, Fuad Ahmad Siddiqi, Laila Yaseen, Kanaz Ahmad Siddiqi, Nidda Yaseen, Imran Khan, Amna Ashraf, Kanza Khalid, Muhammad F Shahid, Naveed Abbas

**Affiliations:** 1 Haematology, Armed Forces Institute of Pathology, Rawalpindi, PAK; 2 Medicine, Combined Military Hospital, Rawalpindi, PAK; 3 Obstetrics and Gynecology, Combined Military Hospital, Rawalpindi, PAK

**Keywords:** folate deficiency, serum vitamin b12, non-megaloblastic, megaloblastic anemia, macrocytosis

## Abstract

Objective

The objectives of this study were to determine the frequency of the clinical spectrum of diseases in patients with macrocytosis and to summarize the diagnostic evaluation of patients found to have macrocytosis on laboratory testing.

Background

This was a cross-sectional study that took place at the Department of Medicine in Combined Military Hospital, Rawalpindi, Pakistan, from January to June 2023.

Methodology

One hundred and five patients with macrocytosis with mean corpuscular volume (MCV) values > 100 fL (80 to 100 fL) were inducted as per inclusion and exclusion criteria. Informed consent was obtained from all patients. Complete blood counts (CBC), peripheral blood film, serum vitamin B12 levels, serum folate levels, renal function tests (RFTs), liver function tests (LFTs), and thyroid function tests (TFTs) were performed during the assessment.

Results

The commonest cause of macrocytosis was vitamin B12 deficiency followed by folate deficiency, combined vitamin B12 and folate deficiency, and other causes were also found in a few cases.

Conclusion

Serum vitamin B12 and folate deficiency are the most common preventable causes of macrocytosis.

## Introduction

Anemia is a frequently faced problem in primary healthcare settings. According to World Health Organization (WHO) criteria, anemia refers to hemoglobin less than 13g/dL in males and less than 12g/dL in females [1]. Macrocytosis is defined as mean corpuscular volume (MCV) greater than 100 fL in adults. Macrocytosis associated with or without anemia is a frequently faced laboratory finding in routinely ordered investigations [2]. It is usually discovered on a routine blood complete picture. The prevalence of macrocytosis is 2% to 4% and males are affected more commonly than females. It is associated with advanced age and 60% of patients have associated anemia [3]. Anemia is classified according to its pathophysiological basis either due to decreased production or increased loss of red blood cells (RBCs) and size of RBCs. Anemia with low MCV is microcytic anemia and is commonly due to iron deficiency anemia or thalassemia. Anemia with high MCV is macrocytic anemia and is commonly due to either megaloblastic anemia or other disorders that may be identified. MCV indicates RBC size on a complete blood picture [4]. Medical practitioners should be aware of the significance of macrocytosis as it might lead to the development of anemia. Macrocytosis signifies a wide range of pathologies. Emphasis should be made on a thorough history including symptoms of anemia (fatigue, generalized weakness, shortness of breath, and palpitations), medication history (hydroxyurea, chemotherapeutic agents, anticonvulsants, and anti-retroviral therapy), surgical history, and dietary habits. General physical examination, systemic examination (pallor, stomatitis, glossitis, tachycardia & neurological signs), and laboratory findings are of paramount importance as they will not only help in identifying the underlying etiology but will also help in making a proper management plan for patients with macrocytosis [3]. During the workup of macrocytosis, serum vitamin B12, folate levels, peripheral blood film, reticulocyte count, liver function tests (LFTs), thyroid function tests (TFTs), and renal function tests (RFTs) play an important role [5]. Vitamin B12 and folate deficiencies are the commonest cause of megaloblastic changes. Vitamin B12 deficiency is common and can affect about 15% of the elderly population. If macrocytic anemia is evaluated properly and the cause especially megaloblastic anemia is identified and treated in time then not only the anemia is resolved but permanent neurological defects caused by its deficiency are also prevented [6]. Treatment of macrocytic anemia needs treatment of the underlying cause. If there is megaloblastic anemia, then vitamin B12 and folate need to be replaced. Oral vitamin B12 and folate supplements along with a diet enriched with vitamin B12 and folate are required [7]. Symptoms of anemia resolve quickly with early treatment [8].

The rationale of this study is to timely identify and evaluate macrocytosis in order to cope with the preventable causes of macrocytosis.

## Materials and methods

This cross-sectional study was conducted at the Department of Medicine, in Combined Military Hospital, Rawalpindi from January to June 2023. The sample size was 105. The inclusion criteria were patients with macrocytosis having MCV > 100 fL, both indoor and outdoor cases, age more than 12 years, and both genders. The exclusion criteria were MCV < 100 fL, patients already taking vitamin B12 and folate supplements, patients who have undergone any abdominal surgery, and those aged 12 years and below. Permission was sought from the ethical committee, after which 105 patients with macrocytosis were inducted via non-probability consecutive sampling. History taking (including dietary history) and clinical examination were carried out in detail. Hospital registration numbers and informed consent were obtained from all patients. Out of 105 patients, 66 were males and 39 were females. Complete blood picture, serum vitamin B12 and folate levels, RFTs, LFTs, TFTs, and peripheral blood smear examination were performed. A blood sample of 3ml was taken for complete blood count and it was analyzed on a Sysmex XN 3000 automated hematology analyzer (Sysmex Corporation, Kobe, Japan). Serum samples of 3 ml were taken for vitamin B12, folate, LFTs, RFTs, and TFTs. Cobas e 801 automated chemistry analyzer (Rocher Diagnostics, Basel, Switzerland) was used for performing LFTs, RFTs, TFTs, vitamin B12, and folate levels. Peripheral blood smear was viewed using a binocular microscope. Vitamin B12 and folic acid deficiency were defined as serum vitamin B12 level <148 pmol/L and folic acid level < 10 nmol/L respectively. IBM SPSS Statistics for Windows, Version 22 (Released 2013; IBM Corp., Armonk, New York, United States) was used for data analysis. Means and standard deviations were calculated for quantitative data like age, hemoglobin, MCV, serum vitamin B12, and folate levels. The qualitative data like gender, deranged vitamin B12, folate, LFTs, RFTs, TFTs, and morphological abnormalities on peripheral smear were analyzed by frequencies.

## Results

A total of 105 patients were included. Sixty-six (62.9%) were males and thirty-nine (37.1%) were females (Table 1). Twenty patients out of 105 were below 35 yrs, 11 patients were between 35 and 44 yrs, 26 patients were between 45 and 54 yrs, 23 patients were between 55 and 64 yrs, 11 patients were between 65 and 74 yrs and 14 patients were above 75 yrs (Table 2). The mean age of the sampled patients (n=105) was 52.2±18.1 years with a minimum of 13.00 and a maximum of 87.00 years, mean hemoglobin was 10.6±2.73 g/dL with a minimum of 5.00 and maximum of 18.00 g/dL, mean MCV was 104.7±5.75 fL with minimum 101 and maximum 126 fL, the mean serum vitamin B12 level was 172.6±112.8 pmol/L with a minimum of 83.00 and a maximum of 380.00 pmol/L and mean serum folate levels were 11.4±3.44 nmol/L with minimum 8.00 and maximum 20.00 nmol/L (Table 3). Anemia was observed in 71.4% of cases (Table 4). Vitamin B12 deficiency was identified as the etiological factor in 57.1% of cases, folate deficiency was identified in 17.1% of cases and combined vitamin B12 and folate deficiency was identified in 10.4% of cases. Other causes identified were chronic liver disease 2.8%, chronic renal failure 1.9%, hypothyroidism 1.9%, and other hematological issues were present in 3.8% of cases. In 5% of cases, no cause was identified (Table 5). The P value was < 0.001 for vitamin B12 and 0.043 for serum folate levels respectively, both being less than α (alpha = 0.05), showing that our results were significant according to the ANOVA (analysis of variance) test applied to our data; moreover, F-statistic was 5.385 for vitamin B12 and 1.132 for serum folate levels. We found that vitamin B12 deficiency has a significantly higher frequency among all the causal factors in patients with macrocytosis. Macrocytosis needs to be investigated either diagnosed with or without anemia as it may be the first clue to the underlying pathology.

**Table 1:** Age distribution

Serial	Age (years)	Frequency	Percent
1.	<35	20	19.0 %
2.	35-44	11	10.5 %
3.	45-54	26	24.8 %
4.	55-64	23	21.9 %
5.	65-74	11	10.5 %
6.	>75	14	13.3 %
Total		105	100.0 %

**Table 2:** Gender distribution

Serial	Gender	Frequency	Percentage
	Male	66	62.9 %
	Female	39	37.1 %
Total		105	100.0 %

**Table 3:** Descriptive statistics MCV: Mean corpuscular volume

Serial	Quantitative Variables	N	Minimum	Maximum	Mean	Standard Deviation
	Age in Years	105	13.00	87.00	52.2190	18.13941
	Hemoglobin g/dL	105	5.00	18.00	10.6476	2.73503
	MCV > 100fL	105	101.00	126.00	104.7429	5.75632
	Serum Vitamin B12 Levels pmol/L	105	83.00	380.00	172.6476	112.83719
	Serum Folate Levels nmol/L	105	8.00	20.00	11.4381	3.44719

**Table 4:** Presence of anemia

Serial	Present / Absent	Frequency	Percent
1.	Present	75	71.4 %
2.	Absent	30	28.6 %
Total		105	100.0 %

**Table 5:** Causes of macrocytosis

Serial	Cause	Frequency	Percent
1.	Vitamin B12 deficiency	60	57.1 %
2.	Folate deficiency	18	17.1 %
3.	Combined vitamin B12 and folate deficiency	11	10.4 %
4.	Other hematological issues on peripheral film	4	3.8 %
5.	Chronic liver disease	3	2.8 %
6.	Chronic renal failure	2	1.9 %
7.	Hypothyroidism	2	1.9 %
8.	Unknown	5	5.0 %
Total		105	100.0 %

## Discussion

Macrocytosis is due to abnormal RBC development, abnormal RBC membrane composition, reticulocytosis, or a combination of all of them. These abnormalities of RBC nuclear maturation and development occur as a result of impaired DNA synthesis. Macrocytic anemia may be megaloblastic or non-megaloblastic. Megaloblastic anemia is associated with abnormal development of RBC. It is most commonly seen in vitamin B12 and folate deficiency. Diet is the source of vitamin B12 and is present in all animal-origin foods such as fish, meat, dairy products, etc, which explains why its deficiency is seen in strict vegetarians [8]. Vegetarian diets are further classified into lactovegetarian (only dairy products in diet), ovovegetarian (only eggs in diet), and lacto-ovovegetarian (both dairy products and eggs in diet). Pernicious anemia also causes vitamin B12 deficiency. Surgeries like gastrectomy will remove the site of intrinsic factor synthesis and ileal surgical resection will eliminate the site of vitamin B12 absorption and will also cause its deficiency. Blind loop syndrome will lead to bacterial overgrowth and competition inhibition of vitamin B12. Vitamin B12 deficiency is also seen in severe Crohn’s disease and prolonged use of proton pump inhibitors and metformin [5]. Folic acid is present in most fruits and vegetables, especially citrus fruits and green leafy vegetables. Folate deficiency may be dietary, due to decreased absorption like in Celiac disease, or due to increased requirement like in pregnancy or chronic hemolytic anemia. Folic acid deficiency is seen in patients taking phenytoin, methotrexate, sulfasalazine, and trimethoprim-sulfamethoxazole [4]. Reticulocytosis is seen in hemolysis, blood loss, or transiently during the recovery phase once the cause of anemia is corrected. Non-megaloblastic causes include hypothyroidism, pregnancy, chronic liver disease, chronic kidney disease, and hematological disorders like myelodysplastic syndrome and myeloproliferative disorders. Pregnant women especially with a history of neural tube defects and people on anti-epileptic drugs should regularly take folic acid supplementation [9]. In megaloblastic anemia, peripheral blood film shows macro-ovalocytes and hypersegmented neutrophils. On a complete blood picture, macrocytosis is labeled when MCV is more than 100 fL [10]. After laboratory evaluation, macrocytosis may remain unexplained in 10% of cases. These cases require close follow-up with six monthly complete blood pictures as they may develop primary bone marrow disorder or worsening cytopenias later on [11]. The aim of this study was to identify and evaluate the underlying causes of macrocytosis detected in routine investigations. In a similar study, Chang et al. studied hematinic deficiencies in a Taiwan cohort with macrocytosis. It was a case-control study that included 60 patients with macrocytosis. Patients with macrocytosis had a significantly higher frequency of hemoglobin (50.0%), vitamin B12 (40.0%), and folic acid deficiency (5.0%) [1]. Veda et al. found alcoholism as a causative factor in 36.5% of cases, vitamin B12 deficiency in 24.1% of cases, drug-induced in 12.9% of cases, and folate deficiency in 4.4% of cases in a cross-sectional study conducted on a cohort of 178 adult patients with macrocytosis. Anemia was also observed in 53.3% of cases [2]. In another trial, Iqbal et al found vitamin B12 deficiency in 78.5% of cases and folate deficiency in 43.4% of cases in a retrospective cohort study of 220 patients with macrocytic anemia [11]. There are, however, other studies that have observed the contradictory results of causes of macrocytosis. Savage et al. found drug therapy and alcohol and liver disease as the most common causes of macrocytosis in 300 consecutive hospitalized patients in a New York City teaching hospital [9]. McNamee et al. conducted a cross-sectional study involving 1,207 adults and found elevated Gamma-glutamyl transferase (25.0%) and smoking (24.6%) association with macrocytosis rather than alcoholism (6.3%), folate (10.5%) and vitamin B12 deficiency (3.4%) [12]. Mischoulon et al. conducted a study on 223 patients at Massachusetts General Hospital and found that macrocytosis did not predict folate or vitamin B12 deficiencies. Among 39 patients with folate deficiency and 25 patients with low vitamin B12 levels, none had macrocytosis [13]. Stouten et al. conducted a cohort study in the Western part of the Netherlands on 3324 patients and found vitamin B12 deficiency (16.6%), renal anemia (13.0%), alcohol (11.2%), bone marrow disorders (9.7%), and folate deficiency (5.8%) among the classical causes in 115 patients [14]. 

## Conclusions

A comprehensive understanding of macrocytosis involves recognizing its diverse etiology and the importance of diagnostic investigations. In our study, serum vitamin B12 and folate deficiency were the most common preventable causes of macrocytosis. Treatment strategies may range from addressing nutritional deficiencies to managing the underlying health conditions contributing to macrocytosis. Regular medical monitoring is crucial to ensure an effective and tailored approach to the individual's health, emphasizing the significance of collaborative efforts between healthcare providers and patients in maintaining optimal well-being.
